# A Dominant Clone of *Leptospira interrogans* Associated with an Outbreak of Human Leptospirosis in Thailand

**DOI:** 10.1371/journal.pntd.0000056

**Published:** 2007-10-31

**Authors:** Janjira Thaipadungpanit, Vanaporn Wuthiekanun, Wirongrong Chierakul, Lee D. Smythe, Wimol Petkanchanapong, Roongrueng Limpaiboon, Apichat Apiwatanaporn, Andrew T. Slack, Yupin Suputtamongkol, Nicholas J. White, Edward J. Feil, Nicholas P. J. Day, Sharon J. Peacock

**Affiliations:** 1 Mahidol-Oxford Tropical Medicine Research Unit, Faculty of Tropical Medicine, Mahidol University, Bangkok, Thailand; 2 WHO/FAO/OIE Collaborating Centre for Reference & Research on Leptospirosis, Centre for Public Health Sciences, Queensland Health Scientific Services, Brisbane, Australia; 3 National Institute of Health, Nonthaburi Province, Thailand; 4 Medical Department, Udon Thani General Hospital, Udon Thani, Thailand; 5 Department of Medicine, Faculty of Medicine, Siriraj Hospital, Mahidol University, Bangkok, Thailand; 6 Centre for Clinical Vaccinology and Tropical Medicine, Nuffield Department of Clinical Medicine, University of Oxford, Churchill Hospital, Oxford, United Kingdom; 7 Department of Biology and Biochemistry, University of Bath, United Kingdom; Institut Pasteur, France

## Abstract

**Background:**

A sustained outbreak of leptospirosis occurred in northeast Thailand between 1999 and 2003, the basis for which was unknown.

**Methods and Findings:**

A prospective study was conducted between 2000 and 2005 to identify patients with leptospirosis presenting to Udon Thani Hospital in northeast Thailand, and to isolate the causative organisms from blood. A multilocus sequence typing scheme was developed to genotype these pathogenic *Leptospira*. Additional typing was performed for *Leptospira* isolated from human cases in other Thai provinces over the same period, and from rodents captured in the northeast during 2004. Sequence types (STs) were compared with those of *Leptospira* drawn from a reference collection. Twelve STs were identified among 101 isolates from patients in Udon Thani. One of these (ST34) accounted for 77 (76%) of isolates. ST34 was *Leptospira interrogans*, serovar Autumnalis. 86% of human *Leptospira* isolates from Udon Thani corresponded to ST34 in 2000/2001, but this figure fell to 56% by 2005 as the outbreak waned (p = 0.01). ST34 represented 17/24 (71%) of human isolates from other Thai provinces, and 7/8 (88%) rodent isolates. By contrast, 59 STs were found among 76 reference strains, indicating a much more diverse population genetic structure; ST34 was not identified in this collection.

**Conclusions:**

Development of an MLST scheme for *Leptospira interrogans* revealed that a single ecologically successful pathogenic clone of *L. interrogans* predominated in the rodent population, and was associated with a sustained outbreak of human leptospirosis in Thailand.

## Author Summary

A sustained outbreak of human leptospirosis occurred in northeast Thailand between 1999 and 2003, the basis for which was unknown. Leptospirosis is a potentially serious infection cause by bacteria known as *Leptospira*; infection usually occurs following environmental exposure to pathogenic *Leptospira* shed in the urine of an infected animal. The purpose of this study was to obtain bacterial isolates from humans with leptospirosis around the time of the Thai outbreak for genotyping, and to relate these to the maintenance host animal. To achieve this, a bacterial typing scheme (multilocus sequence typing, MLST) was developed for *L. interrogans*, the major cause of human disease. This approach has the advantage over existing typing schemes in that the data generated are amenable to detailed evolutionary analysis, and are readily comparable via the internet. Our results demonstrated the emergence of a dominant clone of *L. interrogans* serovar Autumnalis; this was the major cause of human disease during the outbreak, and was found in a maintenance host which was defined as the bandicoot rat.

## Introduction

Leptospirosis is a zoonotic infection caused by pathogenic members of the genus *Leptospira*. Human disease is usually acquired following environmental exposure to *Leptospira* shed in the urine of an infected animal [1],[2]. Infection is acquired during occupational or recreational exposure to contaminated soil and water, organisms gaining entry to the accidental human host via abrasions or less commonly the conjunctiva [1]. Disease may also be acquired through direct contact with infected animals, and occurs in farmers, veterinarians and abattoir workers [1]. The disease has a worldwide distribution but is most common in tropical regions where incidence peaks during the rainy season [1],[2]. Clinical manifestations are broad ranging and follow a biphasic pattern in which a septicemic phase lasting around one week is followed by an immune phase during which antibodies are raised and organisms localize in tissues and appear in urine. Much disease is sub-clinical or mild, but patients reaching medical attention usually have an acute febrile illness associated with one or more of chills, headache, myalgia, conjunctival suffusion, and abdominal symptoms which can include nausea, vomiting and diarrhea [1]. Leptospirosis has been described as anicteric or icteric; the former represents 85–90% of cases and is associated with a good prognosis, while the latter may be associated with multisystem disease involving particularly the kidneys, lung and heart, with a reported mortality rate of 5–15% [1].

Leptospirosis is an emerging infectious disease in Thailand [3],[4]. Before 1996, the number of cases reported to the Department of Disease Control (DDC) was approximately 200 per year. Leptospirosis was sporadic and reported mainly from central and southern regions. A marked change occurred in the subsequent decade, with a year-on-year rise from 398 cases in 1996 to a peak of 14,285 cases in 2000. This was followed by a continuous decline with 2,868 cases reported during 2005 [5]. Reporting in Thailand is voluntary and probably represents a small proportion of true cases. There was also a shift in the geographical distribution, with the majority of cases being reported in the northeast. One explanation for the outbreak is that it was related to the emergence of a biologically successful clone of *Leptospira*. This possibility is supported by a study of 44 leptospiral strains obtained from humans during three outbreaks in Brazilian urban centers, in which typing using arbitrarily primed PCR demonstrated that 43 isolates exhibited very similar fingerprints suggestive of a clonal population of *L. interrogans* [6]. In addition, during a large urban outbreak in Brazil, *L. interrogans* serovar Copenhageni was isolated from 87% of cases with positive blood cultures [7]. Although it is currently unclear to what extent genetic relatedness can be informed by serotype alone, this observation is consistent with the majority of cases being caused by the expansion of a single outbreak clone.

The aim of this study was to define the molecular epidemiology of *Leptospira* strains isolated from humans during the Thai outbreak, and to relate this to the maintenance animal host. To achieve this, an MLST scheme was developed for *L. interrogans*, the major cause of human disease. This approach has the advantage over existing typing schemes in that the data generated are amenable to detailed evolutionary analysis. MLST data are also readily comparable via the internet, and establishment of an MLST scheme therefore paves the way for future studies. Our results confirm the emergence of a dominant clone of *L. interrogans* serovar Autumnalis; this was the major cause of human disease, and was found in a maintenance host which was defined as the bandicoot rat.

## Methods

### Patients and bacterial strains

A prospective study was undertaken at Udon Thani General Hospital in northeast Thailand to identify patients with leptospiremia. This 1,000 bed provincial hospital serves a predominantly rural population, >80% of whom are rice farmers and other agricultural workers who are repeatedly exposed to rats and water contaminated by rat urine. Patients were recruited during consecutive months from October 2000 to December 2002, then for four months during each rainy season (July to October inclusive) during 2003 and 2005. The reason for this pattern of recruitment is that leptospirosis is predominantly a rainy season disease. Consecutive adult patients (≥15 years) presenting with fever (>37.8°C) of unknown cause were recruited following informed and written consent. Patients with a blood smear positive for malaria parasites or other definable infections such as pneumonia or urinary tract infection were excluded. The clinical features of leptospirosis are broad ranging and similar to other acute febrile illnesses common to this geographic area such as scrub typhus and dengue fever. In view of this, all adult patients presenting with acute undifferentiated fever were cultured to detect leptospiremia. A 10 ml blood sample was drawn on the day of admission into a sterile tube containing 250 units of sodium heparin for *Leptospira* culture. The study protocol was approved by the Ethical Committee of the Ministry of Public Health, Royal Government of Thailand.

A further 24 unselected isolates cultured from the blood of patients with leptospirosis presenting to hospitals in 8 additional provinces in Thailand during the rainy seasons of 2003 and 2004 were obtained from strain collections. These provinces were: Lumpang (situated in the north), Yasothon, Nakhon Ratchasima, Maha Sarakahm and Loei (northeast), Ratchaburi (central), Rayong (east) and Chumphon (south).

Seventy six reference strains representative of the species *L. interrogans*, *L. kirschneri* and *L. borgpetersenii* were obtained from the WHO/FAO/OIE Collaborating Center for Reference & Research on Leptospirosis, Australia, or National Institute of Health, Thailand.

### Isolation of *Leptospira* from the maintenance host

A total of 1,126 rodents were trapped in Nakhon Ratchasima, northeast Thailand during 2004 by the National Institute of Health, Thailand. Animals were identified and cultured for *Leptospira*, as described previously [8]. Ten animals were culture positive (*Bandicota indica* 8, *Bandicota savilei* 1, and *Rattus rattus* 1), while all samples from *Rattus exulans, Rattus losea, Mus cervicolor, Mus caroli* and *Sancus murinus* were culture negative for *Leptospira*. Eight unselected isolates remained viable and were evaluated in this study; of these, 6 were isolated from *B. indica* (greater bandicoot rat) and 1 each was isolated from *B. savilei* (lesser bandicoot rat) and *Rattus rattus* (black rat).

### *Leptospira* culture and species identification

Culture of leptospires from human blood was performed using EMJH supplemented with 3% rabbit serum and 0.1% agarose, as described previously [9]. Positive cultures were sent to the WHO/FAO/OIE Collaborating Center for Reference & Research on Leptospirosis, Australia for serovar identification using the cross agglutinin absorption test (CAAT) [10]. Definitive identification of species was undertaken by amplification and sequencing of the near full-length 16S rRNA gene. Primers were designed to anneal to conserved regions of genes from pathogenic species *L. interrogans*, *L. kirschneri*, *L. borgpetersenii, L. santarosai*, *L. alexanderi* and *L. fainei*. The primers (f - 5′ GTTTGATCCTGGCTCAG 3′ and r -5′CCGCACCTTCCGATAC 3′) amplified a 1,483 bp PCR product which was sequenced in its entirety using internal primer pairs (primers available on request).

### Multilocus sequence typing

Genomic DNA was extracted using the High Pure PCR Template Preparation Kit (Roche Applied Science, Germany). In a pilot study, 14 housekeeping loci were selected using the whole genome sequence of *L. interrogans* serovar Lai strain 56601 (11 loci situated on chromosome I and 3 loci on chromosome II; loci and primer sequences available on request). Primers were designed using PrimerSelect software (DNASTAR Inc., Wisconsin,USA), and synthesized by Sigma-Proligo (Proligo Singapore Pty Ltd). These were evaluated using 30 clinical or reference strains belonging to species *L. interrogans*, *L. kirschneri* or *L. borgpetersenii,* using standard MLST methodology [11] (data not shown). Each of the 14 gene fragments were amplified by PCR, purified and sequenced using a MegaBACE 500 sequencer and DYEnamic ET Dye Terminator Cycle Sequencing Kit (Amersham Biosciences, England). Seven loci were then selected based on performance of primers, number of alleles at a given locus and distribution of strain numbers between the alleles. These loci were *pntA*, *sucA*, *fadD*, *tpiA*, *pfkB*, *mreA*, & *glmU*, which are located on chromosome I with the exception of *fadD*. Primer sequences are shown in Table 1. Amplifications were performed in 25-µl total volumes of PCR reaction mix contained 1–10 ng of genomic DNA, 5 pmol of each primer, 200 µM dNTP, (eppendorf, Germany), 1.5 mM of MgCl_2_, 1.25 unit of Taq DNA polymerase (Promega, USA) and 1× buffer. A PTC-200 Peltier Thermal Cycler (MJ research, USA) was used to perform PCR with an initial denature step at 94°C for 5 minutes, followed by 30 cycles of 94°C for 10 seconds, 52°C (*mreA*, *pfkB*, *pntA*, *sucA*, and *tpiA*), or 50°C (*fadD* and *glmU*) for 15 seconds, 72°C for 50 seconds, then 72°C for 7 minutes. PCR product size ranged from 555 bp to 638 bp; the sequence start and end points used to define each MLST locus are shown in Table 1. MLST was performed for the remaining isolates using these 7 loci. Following the standard MLST protocol, each allele was assigned a different allele number and the allelic profile (string of seven integers) was used to define the sequence type (ST). A leptospira mlst website was established to provide public access to these data, and to provide a resource to other investigators who can use this to assign the ST of further strains. This can be accessed at http://leptospira.mlst.net.

**Table 1:** MLST primers and allele frequency in 204 *Leptospira* isolates.

Gene*	Function	TIGR Cellular role category	Primer location	Primer sequence (5′ - 3′)	Location of sequence used to define MLST locus	Number of alleles
pntA	NAD(P) transhydrogenase subunit alpha	Energy metabolism: Electron transport	56283-56301	f-tgccgatcctacaacatta	56347-56871	23
			56899-56920	r-aagaagcaagatccacaactac		
sucA	2-oxoglutarate dehydrogenase decarboxylase component	Energy metabolism: TCA cycle	1227434-1227455	f-agaagaggccggttatcatcag	1227474-1227920	15
			1227993-1227973	r-cttccgggtcgtctccattta		
pfkB	Ribokinase	Energy metabolism: Sugars	1386512-1386531	f-ccgaagataaggggcatacc	1386553-1386984	33
			1387071-1387050	r-caagctaaaaccgtgagtgatt		
tpiA	Triosephosphate isomerase	Energy metabolism: Glycolysis/gluconeogenesis	1694753-1694732	f-aagccgttttcctagcacattc	1694673-1694248	24
			1694199-1694220	r-aggcgcctacaaaaagaccaga		
mreA	Rod shape-determining protein rodA	Cell envelope: Biosynthesis and degradation of murein sacculus and peptidoglycan	2734622-2734601	f-gtaaaagcggccaacctaacac	2734550-2734116	21
			2734021-2734040	r-acgatcccagacgcaagtaa		
glmU	UDP-N-acetylglucosamine pyrophosphorylase	Cell envelope: Biosynthesis and degradation of surface polysaccharides and lipopolysaccharides	3785017-3784999	f-ggaagggcacccgtatgaa	3784955-3784512	18
			3784461-3784479	r-tccctgagcgttttgattt		
fadD	Probable long-chain-fatty-acid–CoA ligase	Not known	83621-83600	f-agtatggcgtatcttcctcctt	83570-83115	19
			83045-83066	r-ttcccactgtaatttctcctaa		

* all loci are situated on chromosome I of *L. interrogans* serovar Lai strain 56601 with the exception of *fadD* which is on chromosome II.

### Nucleotide sequence accession numbers

DNA sequences for the 16S rRNA gene have been deposited in the GenBank database with the accession numbers shown in Table S1.

## Results

### An outbreak of leptospirosis in Thailand

**Figure 1:**
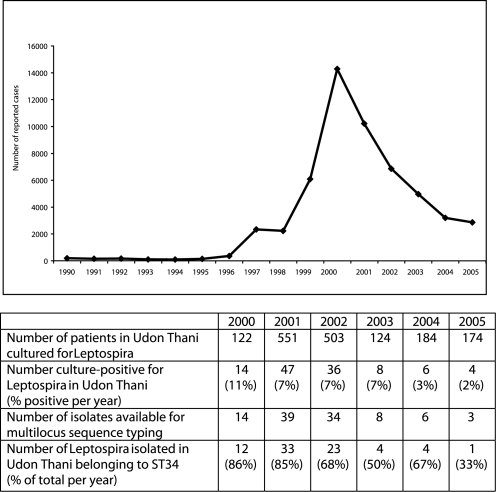
Change in the number of cases of human leptospirosis in Thailand over time. Graph shows the number of leptospirosis cases reported to the Department of Disease Control, Thailand, 1990–2005 (top). (Source: Disease Notification Report, Ministry of Public Health, Thailand). Table shows the number of patients presenting to Udon Thani Hospital with undifferentiated fever who were recruited into a prospective study to define patients with leptospiremia, together with the proportion each year who were culture positive. The number of strains that were evaluated using MLST and defined as sequence type (ST) 34 is also given.

The number of leptospirosis cases reported to the Department of Disease Control, Thailand between 1990 and 2005 is shown in Figure 1. An increase in cases of leptospirosis was also observed by clinicians working in northeast Thailand during 1999 (personal communication, Dr R. Limaiboon, Udon Thani Hospital). A prospective study was commenced at Udon Thani Hospital in mid-October 2000 to identify and culture suspected cases and isolate the causative *Leptospira*.

**Figure 2:**
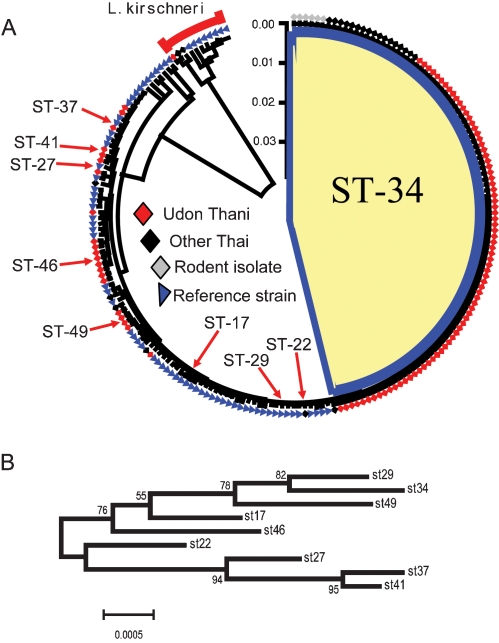
Phylogenetic analysis of 204 *Leptospira* isolates. All isolates are *L. interrogans* except those belonging to the *L. kirschneri* clade, as indicated. Figure 2a. The tree was constructed using the Neighbour-Joining method (K-2-P) as implemented in MEGA ver 3.1 [12]. The numerical dominance of ST34 is indicated by the shaded area. Isolates recovered from Udon Thani are represented throughout the tree (red diamonds). The highlighted STs are discussed in the text. Figure 2b was constructed using MEGA ver 3.1 [12], and shows a neighbour-joining tree based on these highlighted STs only in order to clarify the branching order. Confidence in the topology of this tree was gauged by bootstrap resampling (1,000 times), and the scores are shown on the nodes.

### Prospective identification of patients with leptospiremia in northeast Thailand

A total of 1,658 patients were recruited in Udon Thani, of whom 115 were culture positive for *Leptospira*. The number of cases of culture proven leptospirosis was greatest during 2001 (there were only 2 study months during 2000), followed by a decline to the end of the study in 2005 (Figure 1). There was a significant reduction over time in the proportion of patients presenting with fever who were leptospiraemic (chi-squared for trend = 15.3, p<0.0001). This case load pattern mirrors the number of cases reported to the Department of Disease Control. Our data provides additional confirmatory evidence for a true increase in leptospirosis in northeast Thailand during the putative outbreak.

### Dominance of a single clone of *L. interrogans* ST34 among pathogenic *Leptospira* isolated in Udon Thani

The pathogenic strains of *Leptospira* obtained from patients presenting to Udon Thani hospital were characterized to determine whether the increased disease incidence was related to one or a number of different circulating bacterial clones. Of the 115 isolates obtained from patients in Udon Thani, 104 were available for molecular characterization. 16S rRNA sequencing was performed on at least one representative of each ST. One hundred isolates were *L. interrogans*, 3 were *L. borgpetersenii* and 1 was *L. kirschneri* (Table S1). Three strains (*L. borpetersenii* serovar Javanica) failed to amplify at five or six MLST loci but were identical to each other at *glmU*; these strains are not considered further. The 101 isolates from Udon Thani corresponded to 12 STs but a single ST predominated, with ST34 accounting for 77 (76%), all of which were identified as serovar Autumnalis. Of the remainder, 8 isolates belonged to ST46, 4 isolates were ST49, and the remaining nine sequence types consisted of one or two isolates (Figure 2a, Table S1). A single isolate defined as ST41 was also serovar Autumnalis but this strain is unrelated to ST34, showing divergence at all seven alleles. Thus two strains sharing the same serovar can be distantly related [1], possibly because serovars may arise independently in differently lineages by evolutionary convergence, or by horizontal gene transfer.

To explore the role of the dominant clone ST34 in the putative outbreak of leptospirosis, the proportion of *Leptospira* ST34 was determined for each year of the study in Udon Thani (Figure 1). This demonstrated that the dominance of ST34 declined over time, being replaced by a range of other sequence types (Table 1). The proportion of clinical *Leptospira* isolates that were ST34 fell from 85% in 2000/2001 to 64% in 2002/2003 and 56% in 2004/2005 (years combined because of small numbers in 2004 and 2005 - χ^2^ for trend = 6.61, p = 0.01).

### Dominance of ST34 as a cause of leptospirosis across Thailand

To define the extent to which ST34 was distributed across Thailand, a further 24 unselected isolates obtained in 2003 and 2004 from human cases of leptospirosis from across the country were evaluated. The total proportion of isolates corresponding to ST34 was 17/24 (71%) (two strains were non-typable *L. borpetersenii*). The geographic distribution was as follows: Lumpang, 1/2 isolates; Rayong, 1/1 isolate; Chumphon, 2/4 isolates; Loei, 9/9 isolates; Ratchaburi, 1/1 isolate; Yasothon, 1/1 isolate; Nakon Ratchasima, 2/5 isolates; and Maha Sarakham, 0/1 isolate. This is not significantly different from the proportion of ST34 in isolates from Udon Thani in the same years (Fisher's exact p = 0.37), and confirms that the outbreak clone ST34 was widely distributed throughout Thailand and formed the predominant virulent strain at the time of the outbreak. A further six STs were identified in this collection, four of which were not observed in the Udon Thani collection. These data provide further support for the picture of a single dominant clone (ST34) associated with an increased incidence of human disease, within a “background” population of higher genotypic diversity. One strain (ST22 obtained in Lumpang province) was serovar Autumnalis, but showed divergence at 5/7 alleles from ST34.

### The bandicoot rat as a maintenance host

To determine whether a link could be identified between ST34 and a maintenance host, 8 isolates available from rodents captured in northeast Thailand were characterized. Seven strains (from *B. indica* (6) and *B. savilei* (1)), were *L. interrogans* ST34. This confirms the predominance of the outbreak strain in a maintenance host, which in this case appears to be the bandicoot rat. The remaining isolate from *R. rattus* was *L. interrogans*, ST49 which was also isolated from human cases in Udon Thani in 2001/2 (n = 4) and Nakhon Ratchasima in 2004 (n = 1). This does not exclude the possibility of additional maintenance hosts, but rodents trapped in agricultural areas reflect the species to which farmers are commonly exposed.

### Thai isolates are clonally restricted compared with reference collections

To place the Thai isolates within a global context, we selected a total of 76 reference strains representative of the species of the *Leptospira* strain population in Thailand but recovered from diverse geographical sources (*L. interrogans* 65, *L. borgpetersenii* 3, *L. kirschneri* 8) (Table S1). From our Thai sample of 123 clinical isolates, 16 STs were identified (0.13 ST per isolate; 5 strains of *L. borgpetersenii* being non-typable by MLST). In contrast, MLST revealed 59 STs for 73 reference strains (0.81 ST per strain), revealing that the reference strains are far more diverse, and that only a small fraction of the global diversity was recovered in the Thai sample. The reference *L. borgpetersenii* strains did not amplify at all seven loci, and so are again scored as non-typable. The largest clones within the reference collection were ST17 and ST37 (both with 4 isolates); one ST contained 3 isolates, 7 STs contained two isolates and the remaining 49 sequence types had one representative strain (Figure 2a, Table S1). ST34 was not represented in the reference collection. One strain was serovar Autumnalis (Akiyami A, ST27) but this was unrelated to ST34 and was much more similar to the non-ST34 Autumnalis strain isolated in Udon Thani (ST41). This analysis indicates that the strains causing human disease in Thailand are more clonally restricted than reference strains from variable hosts and geographical locations, and that the population genetic structure of *L. interrogans* is highly diverse when considering non-ST34 isolates. This further supports the argument that the predominance of ST34 during the Thai outbreak does not reflect a clonal population structure, and is consistent with a temporary selective advantage.

### Phylogenetic analysis of *L. interrogans* ST34

A phylogenetic analysis was performed to shed light on the emergence of ST34. All 204 typable strains (excluding 8 non-typable *L. borgpetersenii* isolates) were evaluated to identify the close relatives of ST34. Figure 2 shows two neighbour-joining trees based on the concatenated sequences of the seven MLST genes (3165-bp). Figure 2a was constructed using all 204 isolates. There was a clear distinction between the two species *L. interrogans* and *L. kirschnerii* which was also noted in loci individually (not shown). ST34 isolates accounted for almost half of the tree, illustrating the numerical dominance of this clone. As the branching order of this tree is unclear, Figure 2b shows a neighbour joining tree for just the STs highlighted in Figure 2a. Of the four Autumnalis STs, ST27 and ST41 appear closely related in both Figure 2a and 2b, but unrelated to the other Autumnalis STs ST22 and ST34. This latter pair appears to be closely related in Figure 2a, but Figure 2b reveals this is an artifact of the poorly resolved topology of this tree. The different clones sampled from Thailand in this study did not form a single cluster but were dispersed throughout the tree. This suggests that they have not all diverged from a single common Thai ancestor. The lack of evidence for strong geographical structure is consistent with high rates of migration via the rodent (or possibly human) host. Figure 2b identifies ST29 (reference strain Bangkinang 1) as a close relative of ST34; this was isolated from a human in Indonesia. Other close relatives of ST34 are also reference strains from Indonesia and Malaysia (not shown), although the significance of this is unclear as the tree is not robustly supported.

## Discussion

Human outbreaks of leptospirosis are well documented in the literature, as are clusters of cases linked by specific water-related activities or occupations [1],[2]. Outbreaks in Thailand and elsewhere are often linked to climatic events such as flooding and the concomitant increase in human exposure to environments contaminated by *Leptospira*. The precipitous increase in reported cases of leptospirosis in Thailand commencing in 1999, followed by the sustained incidence during the ensuing years, could not be explained by persistent climatic change or sequential episodes of regional flooding. Changes in reporting practice can lead to marked changes in the perceived disease incidence, although this does not explain the marked rise and fall in reported cases over time. An alternative explanation is that this was associated with the presence of a biologically successful clone of pathogenic *Leptospira*. In this study, we developed and applied robust typing methods to provide several lines of evidence in support of this hypothesis. This clone is likely to harbour an adaptive (competitive) advantage, albeit transiently. Possible explanations include a selective advantage for ST34 in the maintenance host (the bandicoot rat) leading to a higher bacterial load and higher shedding from urine, or a survival advantage once shed into environment, such as increased resistance to desiccation. Both possibilities are amenable to testing in the laboratory setting. Alternatively, ST34 may have a greater propensity to cause human disease compared with other circulating clones. Although difficult to test, the finding that ST34 co-existed in the environment with a large number of other STs but caused most disease would be supportive of this hypothesis. The virulence of ST34 as reflected by severity of human disease was not assessed in patients presenting to Udon Thani hospital, since the comparator group was small and caused by 11 other STs. The emergence of ST34 may have predated the outbreak, and this is difficult to refute since no strains were available from the period prior to the outbreak. However, the decline in frequency of ST34 as a cause of leptospirosis over time is consistent with the suggestion that there is a direct link between the clone and the outbreak.

Previous studies of human outbreaks have largely relied on serological methods to confirm clinical cases and to define indirectly the infecting isolate [1]. The standard serological method (microscopic agglutination test, MAT) provides a broad idea of serogroups responsible for leptospirosis in a given geographic area, but in one study the predominant serogroups at a titer of ≥100 correctly predicted less than 50% of serovars [13]. Arbitrarily primed PCR has been used successfully to study human outbreaks in Brazil [6], and to characterize 40 isolates recovered from humans between 1995 and 2001 on the Andaman and Nicobar Islands in India, 32 of which were a clone with a fingerprint matching that *of L. interrogans* sensu stricto [14]. Here, we use the more discriminatory and robust method of MLST to identify clusters of closely related isolates. The use of multiple gene loci is essential, as frequent recombination within the population would make inferences based on single gene loci unreliable [15]. This study clearly demonstrates the advantages of bacterial isolation in that it permits detailed typing studies to characterize local populations and outbreaks.

The MLST scheme presented here was developed primarily to characterize the isolates responsible for the outbreak of leptospirosis unfolding in Thailand in the early 2000s (i.e. *L. interrogans* and the closely related L. *L. kirschneri*), and is not designed for the characterization of the genus as a whole. Nevertheless, the scheme presented here demonstrates the utility of MLST for *Leptospira* for characterizing isolates from a clinical perspective. For more taxonomic or genus-wide evolutionary studies, or for disease caused by other *Leptospira* species, the primer sequences could be refined in order to broaden the phylogenetic range over which they amplify, or alternatively the loci used by Ahmed *et al*. may be employed [16].

In conclusion, our observations provide strong support for the hypothesis that the ST34 clone was associated with the 1998–2003 outbreak of leptospirosis in northeast Thailand. The existence of this strain collection now provides a unique opportunity to study the basis for pathogenicity and disease acquisition.

## Supporting Information

Table S1 Strain details and MLST results for *Leptospira* spp. included in this study
(0.05 MB XLS)
